# Whole genome sequencing for mutation discovery in a single case of lysosomal storage disease (MPS type 1) in the dog

**DOI:** 10.1038/s41598-020-63451-4

**Published:** 2020-04-16

**Authors:** Tamer A. Mansour, Kevin D. Woolard, Karen L. Vernau, Devin M. Ancona, Sara M. Thomasy, Lionel Sebbag, Bret A. Moore, Marguerite F. Knipe, Haitham A. Seada, Tina M. Cowan, Miriam Aguilar, C. Titus Brown, Danika L. Bannasch

**Affiliations:** 1 Department of Population Health and Reproduction, School of Veterinary Medicine, University of California, Davis, CA United States; 2 Department of Clinical Pathology, School of Medicine, Mansoura University, Mansoura, Egypt; 3 Department of Pathology, Immunology and Microbiology, School of Veterinary Medicine, University of California, Davis, CA United States; 4 Department of Surgical and Radiological Sciences, School of Veterinary Medicine, University of California, Davis, CA United States; 5 VCA West Coast Specialty and Emergency Animal Hospital, Fountain Valley, CA United States; 6 Department of Ophthalmology & Vision Science, School of Medicine, University of California, Davis, CA United States; 7 Department of Veterinary Clinical Sciences, College of Veterinary Medicine, Iowa State University, Ames, IA United States; 8 William R Pritchard Veterinary Medical Teaching Hospital, School of Veterinary Medicine, University of California, Davis, CA United States; 9 Department of Pathology and Laboratory Medicine, Brown University, Providence, RI United States; 10 Department of Pathology, Stanford University, Palo Alto, CA United States

**Keywords:** Metabolic disorders, Genetics research, Experimental models of disease, Disease genetics, Genetic testing, Next-generation sequencing, Animal breeding, Neurological disorders

## Abstract

Mucopolysaccharidosis (MPS) is a metabolic storage disorder caused by the deficiency of any lysosomal enzyme required for the breakdown of glycosaminoglycans. A 15-month-old Boston Terrier presented with clinical signs consistent with lysosomal storage disease including corneal opacities, multifocal central nervous system disease and progressively worsening clinical course. Diagnosis was confirmed at necropsy based on histopathologic evaluation of multiple organs demonstrating accumulation of mucopolysaccharides. Whole genome sequencing was used to uncover a frame-shift insertion affecting the alpha-L-iduronidase (*IDUA*) gene (c.19_20insCGGCCCCC), a mutation confirmed in another Boston Terrier presented 2 years later with a similar clinical picture. Both dogs were homozygous for the *IDUA* mutation and shared coat colors not recognized as normal for the breed by the American Kennel Club. In contrast, the mutation was not detected in 120 unrelated Boston Terriers as well as 202 dogs from other breeds. Recent inbreeding to select for recessive and unusual coat colors may have concentrated this relatively rare allele in the breed. The identification of the variant enables ante-mortem diagnosis of similar cases and selective breeding to avoid the spread of this disease in the breed. Boston Terriers carrying this variant represent a promising model for MPS I with neurological abnormalities in humans.

## Introduction

Mucopolysaccharidosis (MPS) is a type of lysosomal storage disease caused by a deficiency of enzymes integral to the breakdown of glycosaminoglycans (GAGs). This serious condition, described in many species including humans and dogs, leads to the accumulation of metabolites of the GAG degradation pathway within lysosomes along with increased urinary excretion of these metabolites^1^. There are 11 known enzymes that regulate the catabolism of four different GAGs. The GAGs affected in each variant of MPS are dependent upon which enzyme is deficient^2^.

In people, MPS is a chronic disease with a progressive course. Neurological abnormalities are a major component of the disease. The progressive cerebral disease typically occurs in MPS III and severe forms of MPS I, II, and VII but chronic hearing loss and vision impairment can occur in all types. Thickening of meninges and diffuse changes to the brain's white matter are also common^3,4^. Most MPS types, except for MPS III, are associated with skeletal abnormalities, joint disease, short stature and abnormal facies^5^. Cardiovascular dysfunctions like valvular heart disease, narrowing of coronary arteries, and eccentric hypertrophy are universal to all subtypes but more common in MPS I, II, and VI^6^. Corneal opacification is a common ocular abnormality in MPS I, VI, VII, less common in MPS IV, and discrete if any in MPS II^7,8^. Other somatic manifestations may include recurrent infections, obstructive airway, enlarged tongue, and hepatosplenomegaly^1,9^. The clinical characterization of disease subtypes may be challenging because of overlap in clinical features and the clinical heterogeneity in each subtype^1,10–13^. Even with the same enzyme affected, different underlying causative mutations have different clinical presentations^14,15^.

Animal models have become extremely important in the continued investigation of MPS disorders^16,17^. Unlike rodent models in which specific genetic mutations are induced, dogs with naturally occurring disease represent an accurate model that better reflect the complex genetic, environmental, and physiological variation present in humans^18^. Several canine models of MPS were characterized. Specifically, MPS types I, II, IIIA, IIIB, VI, and VII have been identified in dogs^19^. Impaired enzymes and accumulated GAGs in different dog breeds perfectly match their corresponding subtypes in human (Supplementary Table S1). Moreover, MPS in dogs recapitulates most of the clinical features in human patients^17^, however some discrepancies have been documented^20,21^. The only canine model of MPS I is described in the Plott Hound breed, with dogs presenting with severe joint and bone disease but no detectable neurologic deficits^22^. Given the heterogeneity of disease subtypes in humans, and the incomplete representation of the whole spectrum of MPS I manifestations in dogs, each new spontaneous mutation in dogs comes with a new golden opportunity to better characterize the disease and utilize canine models for translational research.

MPS is autosomal recessive in dogs, except for MPS II which has an X-linked mode of inheritance^16^. Causative genetic variants were found in coding sequences of several lysosomal enzymes for MPS IIIA^23,24^, MPS VI^25,26^, and MPS VII in dog patients^27–29^. However, MPS I in Plott Hounds was explained by the retention of intron 1 in the alpha-L-iduronidase (*IDUA*) gene caused by a mutation in the donor splice site. Some of the MPS mutations are shared across multiple breeds while others appear to be breed specific^30,31^.

In this work, a Boston Terrier dog was presented to the UC Davis William R Pritchard Veterinary Medical Teaching Hospital (VMTH) with progressive neurological and ophthalmic abnormalities. Urine metabolic screening, bilateral corneal deposits, and multi-organ infiltration of mucopolysaccharides confirmed on necropsy were all highly suggestive of MPS disease. A pipeline for the identification of the causative mutation in a single MPS patient is presented here using whole genome sequencing (WGS). A frame shift mutation in *IDUA* gene caused by an 8-nucleotide insertion was identified as the causative mutation. The mutation was confirmed in a second Boston Terrier dog examined at the VMTH 2 years later. The clinical presentation and pathological analysis of this newly identified sequence variant is described.

## Results

**Figure 1:**
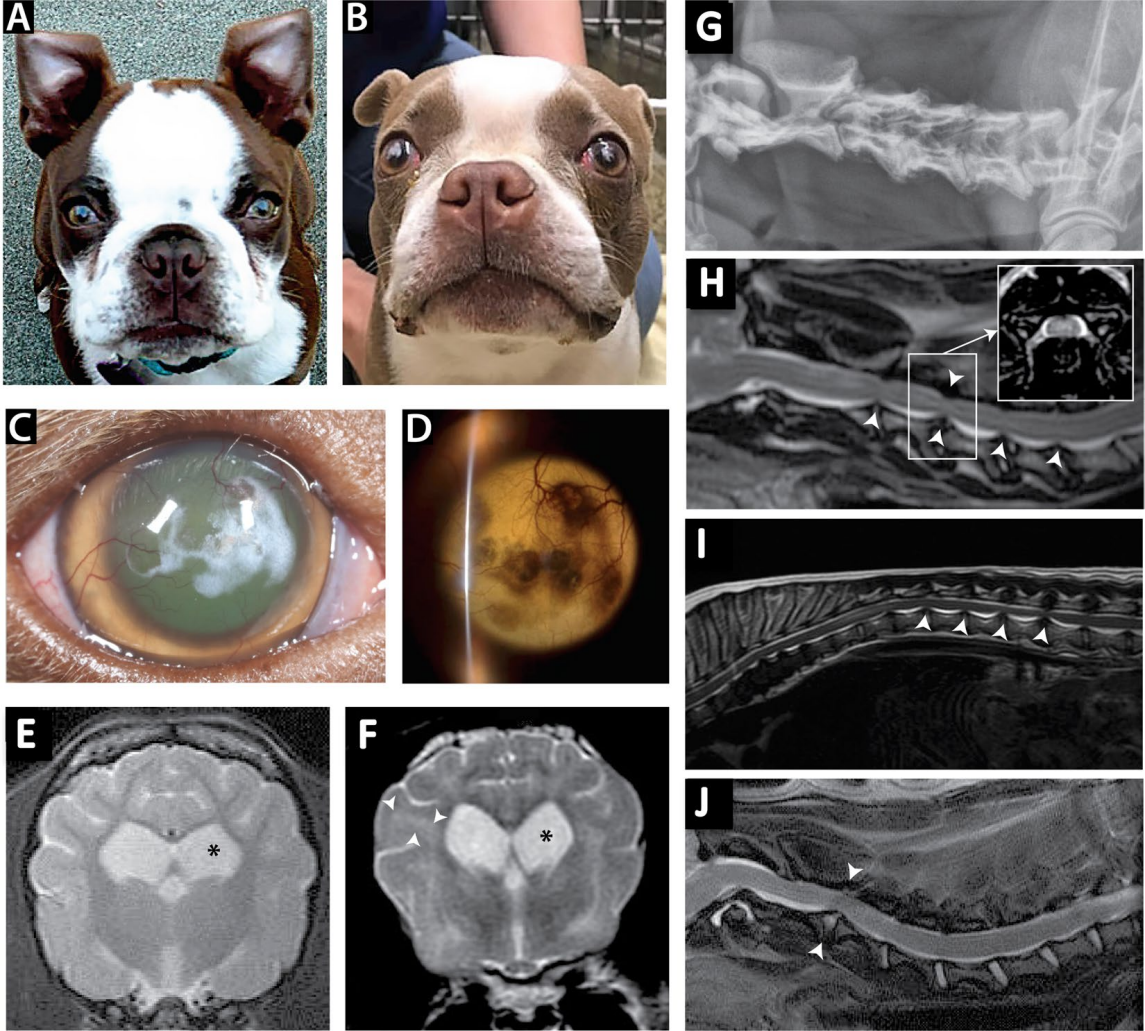
Atypical coat colors, corneal opacities and multifocal central nervous system disease. Two spayed female Boston Terriers age 15 months (**A**) and 21 months (**B**), with atypical chocolate coat color diagnosed with MPS type I. Corneal opacities characterized by white, crystalline stromal infiltrates with direct illumination (**C**) or dark, shadowing regions with retroillumination (**D**) in dog B. Transverse T2 weighted MR images of the brain at the level of the thalamus showed moderate generalized ventriculomegaly, cerebral cortical atrophy and a thin corpus callosum, with hyperintensity of the cerebral white matter and thalamus in dog A (**E**) and dog B (**F**). Lateral radiograph of cervical vertebral column in dog B showed short vertebral bodies, multifocal intervertebral disc space narrowing, ill-defined vertebral end plates and sondylosis deformans (**G**). Sagittal T2 weighted MR images of the cervical (**H**,**J**) and thoracolumbar spinal cord (**I**) showed multifocal protruding intervertebral discs into the spinal canal (arrowheads) with spinal cord stenosis (inset transverse T2 MRI in **H**) in dog A (**J**) and dog B (**H**,).

A 15-month-old female spayed Boston Terrier was presented to the VMTH with a 2-month history of progressive generalized ataxia and abnormal mentation. The dog had been ataxic and lethargic since 4–6 months of age. On physical examination, bilateral grade I/IV medial patellar luxations and bilateral carpal and tarsal hyperextension were noted. Skull conformation was atypical for the patient's age and breed, characterized by a broad and dome-shaped head, wide set eyes and shallow orbits. The coat color was chocolate brown (Fig. 1A), an atypical feature that is not standard according to the Boston Terrier guidelines from the American Kennel Club. Ophthalmic examination revealed mild conjunctival hyperemia, superficial corneal neovascularization, and an approximately 7 ml ill-defined geographical area of crystalline white deposits present in the anterior to mid-corneal stroma axially. Ocular changes were bilateral and symmetrical. Tear production and intraocular pressures were adequate in both eyes. Neuro-ophthalmic examination was unremarkable, with an intact menace response, dazzle, palpebral and pupillary light reflexes. Neurological examination showed mild obtundation, generalized ataxia, and delayed to absent proprioceptive positioning in all limbs. All segmental reflexes and cranial nerve reflexes/responses were appropriate. The patient was reactive upon palpation of the entire vertebral column; however, overt apparent pain was not elicited. These neurological abnormalities were consistent with multifocal central nervous system disease.

Results of the complete blood count, biochemical profile, and urinalysis were unremarkable. Thoracic radiographs showed multifocal intervertebral disc space narrowing with spondylosis deformans and moderate hepatomegaly. On abdominal ultrasound the liver was mildly enlarged and diffusely hyperechoic. On magnetic resonance imaging (MRI) of the brain there was generalized ventriculomegaly (Fig. 1E). An MRI of the cervical spinal cord showed spinal stenosis secondary to a protruding intervertebral disc into the spinal canal (arrowheads) (Fig. 1J). A lysosomal storage disease was the top differential diagnosis for the patient given the dog's young age, slow deterioration of clinical signs, multifocal central nervous system disease, corneal deposits, skull and vertebral column abnormalities, hepatomegaly, and unremarkable blood work. Subsequent urine metabolic screening was positive, suggestive of MPS.

**Figure 2:**
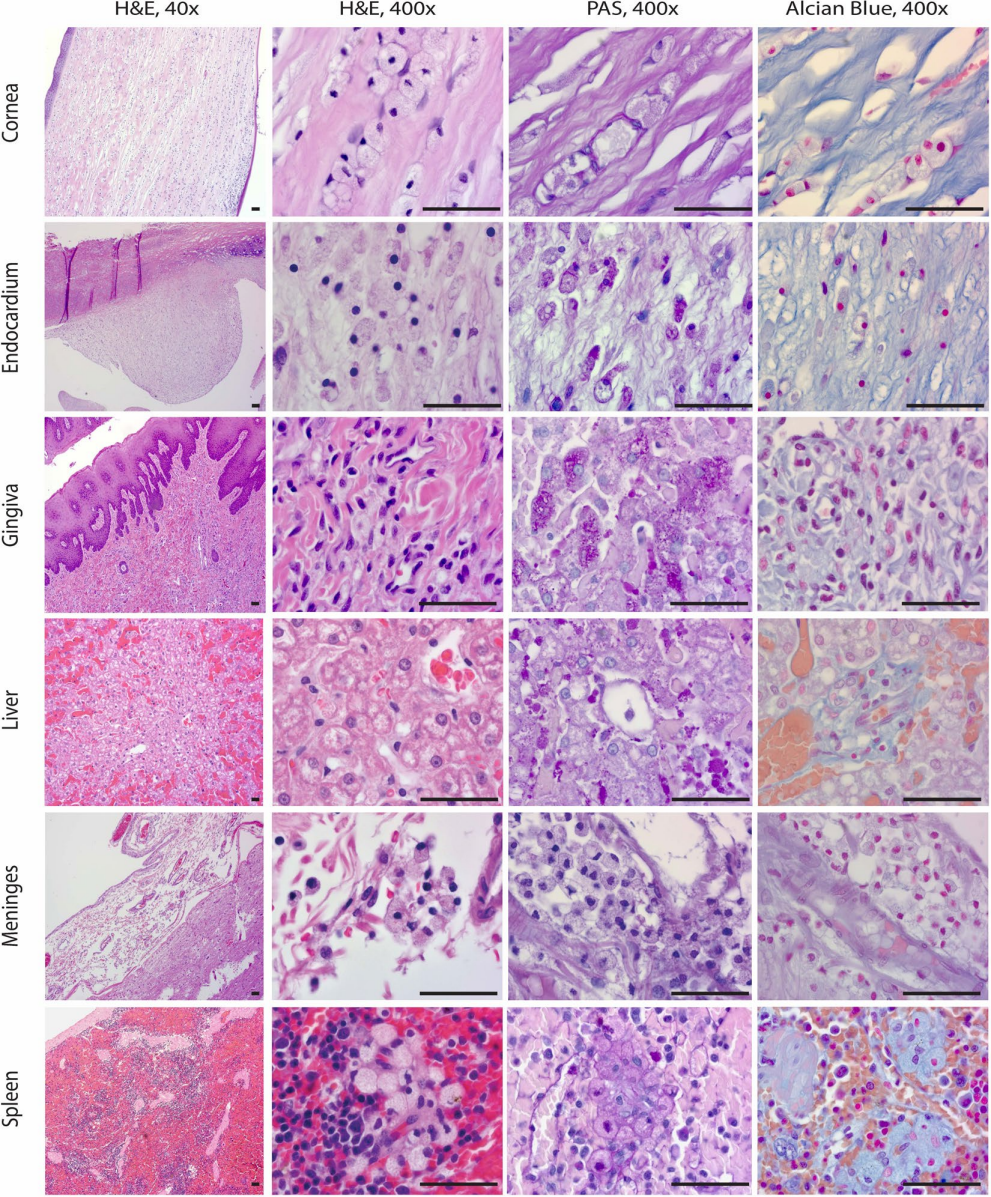
Systematic necropsy examination confirms type I MPS. Each row shows four sections of the examined tissues. The first section is stained by H&E and examined at 40x magnifications. The latter 3 sections are stained by H&E, PAS, and Alcian blue respectively and examined at 400x magnification. Tissue sections show cellular infiltration (H&E 40×). Higher magnification shows that macrophages exhibit abundant, vacuolated cytoplasm (H&E 400×). Macrophages are variably positive on PAS and Alcian blue stains. Bar = 100 micrometers.

Based on worsening clinical signs and poor prognosis, the dog's owners elected for humane euthanasia followed by necropsy. On gross assessment, the corneas were opaque white with prominent small blood vessels. The tongue was globally enlarged with an ulcerated nodule protruding from the left aspect of the tongue base. The liver was slightly enlarged. Within the heart, the septal leaflet of the tricuspid valve was diffusely thickened. The mitral valve was thickened by approximately 15, small, smooth-surfaced nodules present in both valvular leaflets. The aortic valve exhibited similar, nodular thickening. In the brain, the leptomeninges were opaque, obscuring vascular profiles. On cross section of the fixed brain, mild ventriculomegaly was noted within the lateral ventricles. Histopathologic examination of the cornea, endocardium, gingiva, and meninges revealed variably cellular tissue infiltrates composed of macrophages containing abundant, vacuolated cytoplasm. On histochemical stains, cytoplasmic vacuoles within macrophages were positive for periodic acid Schiff (PAS) and Alcian blue stains consistent with glycosaminoglycan accumulation. Within the liver, the hepatic sinusoids were hypercellular, with an increased number of Kupffer cells exhibiting similar cytoplasmic vacuolation and histochemical stain profiles. The splenic white pulp was expanded by nodular aggregates of identically appearing macrophages (Fig. 2).

WGS was performed for unbiased screening of the dog genome using the canFam3 assembly to uncover the underlying genetic change^32^. Approximately 350 million 150 bp paired-end (PE) reads were generated using Illumina sequencing. Approximately 323 million PE reads survived quality filtering, and a 97.14% mapping rate was achieved, yielding 40x coverage of the reference genome. The variant calling pipeline identified 6,168,093 variants in total. To find the causative variant, several steps of filtration were applied based on the incidence of the disease, expected mode of inheritance, and functional annotation of the genomic variants. To exclude common variants in dogs, a control database of common genomic variants found in 100 dogs including 2 Boston Terriers with minor allele frequency >1% was used^33,34^. Only 150,668 variants were found to be unique to the affected dog. As a recessive disease, the causative mutation was expected to be a homozygous or compound heterozygous deleterious coding variant. Functional annotation was done to retain variants with high impact on their proteins’ function according to the NCBI (last modified on 9/5/17) and Ensembl (v98) annotations (Supplementary Table S2). Using NCBI annotations, there were 45 deleterious homozygous mutations affecting 43 transcripts belonging to 36 genes. Also, there were 102 deleterious heterozygous mutations in the genome. However, compound heterozygous inheritance requires at least 2 detrimental mutations affecting both haplotypes of a gene. Only 25 heterozygous mutations affecting 12 genes could fulfil these conditions. According to the NCBI annotations, genes with known genome assembly errors were excluded from the analysis. This filter removed 17 homozygous and 6 heterozygous variants affecting 11 and 3 genes respectively. Among the remaining 28 homozygous variants, only 10 variants affecting 8 genes were annotated to have highly deleterious impact on their protein products based on the Ensembl annotation as well. Also, the remaining 19 heterozygous mutations had only 12 deleterious variants affecting 6 genes according to the Ensembl annotation (Supplementary Table S2).

Further identification of the exact causative gene required a candidate gene approach. There are 11 known MPS causative genes in humans. We examined all possible canine gene annotations (Broad protein tracks, Ensembl and NCBI) to identify the corresponding canine orthologs (Supplementary Table S3). The list of candidate genes was examined for any detrimental homozygous or heterozygous mutations. Only the *IDUA* gene showed precisely one deleterious homozygous frame-shift insertion (c.19_20insCGGCCCCC). Due to the repetitive nature of the locus sequence, the insertion could have happened after any base pair from 3–19, but the effect on the protein sequence remains the same (Supplementary Notes). The insertion in the *IDUA* gene leads to a premature stop, which causes a truncated protein (108 instead of 655 amino acids). The sequence causes a frame-shift after position number 6 and Blastp alignment^35^ fails to find a significant overlap between the two protein products. According to several protein domain databases summarized by Ensembl v91^36^, the mutation abolishes the glycosidase hydrolase domain (Supplementary Notes).

**Figure 3:**
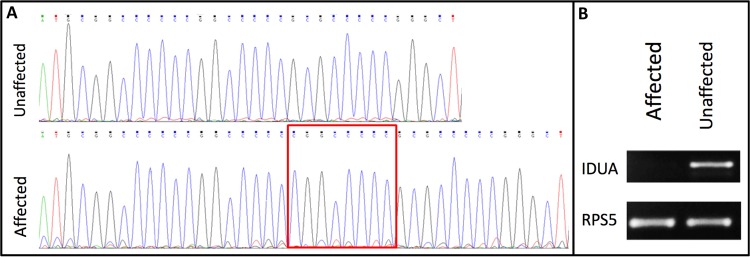
Molecular changes of the *IDUA* gene. (**A**) Sanger sequencing traces of the candidate insertion (c.19_20insCGGCCCCC) in the *IDUA* gene. (**B**) Semi-qRT-PCR of *IDUA* and *RPS5* expression in spleen samples from affected and unaffected Boston Terriers.

The *IDUA* gene insertion was confirmed by Sanger sequencing (Fig. 3A)^37^. To quantify the *IDUA* expression, RT-PCR was performed using RNA extracted from the patient's spleen and a matching sample from another unaffected Boston Terrier. The analysis shows no expression in the patient's sample (Fig. 3B and Supplementary Notes).

To calculate the population allele frequency, Sanger sequencing was used to genotype 120 Boston Terriers as well as 202 dogs from different breeds (Supplementary Table S4). The insertion (chr3  :g.91534556_91534557insGGGGGCCG) was not identified in any samples in either 1 or 2 copies.

Two years after the presentation of the first case, a second female spayed Boston Terrier, with similar clinical signs (abnormal mentation and trouble walking in all four limbs) was presented to the VMTH. She was 21 months old at the time of presentation. On physical examination, she was noted to have a dilute chocolate colored coat, wide set eyes, a markedly domed head, and peg like incisors (Fig. 1B). A grade II/VI basilar systolic heart murmur was audible. The dog had bilateral central white, crystalline corneal deposits in the anterior stroma with superficial vascularization (Fig. 1C,D). Neurological examination showed multifocal CNS disease with mild obtundation, moderate ataxia and tetraparesis. Proprioceptive positioning was absent in all four legs and pain on cervical flexion was apparent.

On cardiac ultrasound, there was moderate aortic valve insufficiency, heterogeneous left ventricular myocardium, and trace mitral valve insufficiency. A serum troponin was normal. On vertebral column radiographs, there was an irregular appearance of the vertebral bodies and end plates of C3–4, C4–5 and T4–8, with collapse of the intervertebral disc spaces and thoracic spondylosis deformans. MRI of the brain showed moderate generalized ventriculomegaly and multifocal cerebral white matter, thalamic, brainstem and spinal cord T2 weighted and FLAIR hyperintensities (Fig. 1F–I), as well as multifocal protruding intervertebral discs into the cervical and thoracolumbar spinal canal with spinal cord stenosis (Fig. 1H,I). The protein of the cerebrospinal fluid sample was mildly elevated (42 mg/dL - normal 0–25 mg/dL), with a slightly elevated total nucleated cell count of 3 cells/uL (normal 0–2 cells/uL). On cytological evaluation, many mononuclear cells contained abundant, large chunky metachromatic granules suggestive of Alder-Reilly bodies. Quantitative analysis of GAGs in urine showed a high level compared to unaffected controls (31.8 mg/mmol creatinine VS 2.9 mg/mmol creatinine). Sanger sequencing showed this case homozygous for the same insertion mutation identified in the first Boston Terrier. Since the two affected dogs had similar and unusual coat colors for the breed, the coat color genes of both dogs were genotyped. The first affected dog was found to be TYRP1^b/b^ and MLPH^D/d^ consistent with its chocolate coat color and the second dog was TYRP1^b/b^ and MLPH^d/d^ consistent with its dilute chocolate coat color

## Discussion

MPS is a debilitating disease affecting humans, dogs and many other species^17^. In this work, we document the clinical presentation of two Boston Terriers with MPS type 1. Whole genome sequencing was used for the discovery of the underlying causative mutation in one of the dogs and the same mutation was confirmed in the second dog.

The first Boston Terrier case was presented with multifocal central nervous system disease, corneal opacities and vertebral anomalies, all suggestive of a lysosomal storage disease. Urinary spot test was positive for MPS, and post-mortem histology of diverse tissues showed large foamy macrophages filled with clear intracytoplasmic vacuoles. Boston Terriers with central nervous system abnormalities described in the present report mimic the clinical manifestations of humans affected by severe forms of MPS I, in which progressive cerebral disease develops with depositions of the GAGs in the meninges and white matter of the brain^3,4^. In contrast, the only other model of MPS type I documented in dogs (Plott Hounds) has an earlier onset of disease causing stunted growth of affected animals^30^. MPS I in Plott Hounds is characterized by severe joint and bone disease with overt pain but no detectable neurologic deficiencies^30^. Lesions in the heart, eyes, and tongue are common to both animal models however the abnormal facies and hepatomegaly seen in Boston Terrier are typical features of the human disease but are not apparent in the Plott Hound dogs.

There are no published variants causing MPS in Boston Terriers despite reports about the disease in the breed^16^. Clinical diagnosis of the first Boston Terrier with MPS at the VMTH motivated the molecular diagnosis of the affected patient to enable the estimation of the mutant allele frequency in the Boston Terrier population. In the absence of suitable family structure or enough cases and controls, techniques like genetic linkage and genome wide association are not useful for disease gene discovery^38,39^. In conditions with a defined list of candidate genes, targeted sequencing is sometimes used^40,41^. However, challenges of custom enrichment, poor uniformity of coverage. and unexpected candidate genes are known disadvantages of this approach^42^. Improved canine exome designs are available and cost efficient however limited by the accuracy of available annotation and restricted for detection of protein coding variants^43^. WGS not only provides extended coverage but also yields better data with more uniform and reliable coverage, less GC and reference biases, and even more power for detecting exome variants^44^. In dogs, WGS has been successfully used in disease gene discovery in a family trio. In the absence of candidate disease genes, additional genotyping of extra cases was needed^45^. WGS was also able to identify the causative coding variant of oculocutaneous albinism in candidate genes^46^.

In this study, WGS was used to identify all genetic variants in a single MPS case. The subsequent bioinformatics pipeline filtered these variants based on the expected mode of inheritance, against a database of canine variants, and according to their expected detrimental effect on the coding sequence. This pipeline was able to reduce the initial 6,168,093 variants to only 10 homozygous mutations and 12 heterozygous mutations (Supplementary Table S2). The known pathophysiology of the disease and the previously identified mutations found in the other MPS cases suggested that the mutation would be in one of the several genes involved in GAGs metabolism^17^. An independent search across candidate genes of enzymes known to regulate the metabolism of GAGs was able to identify the single mutation that overlapped with the list of variants found to be homozygous in this case. The mutation is a frame-shift insertion introducing a premature stop in the *IDUA* gene (c.19_20insCGGCCCCC). The mutation is very likely to be detrimental to the gene's function because it produces a truncated protein. The new protein is not only shorter (108 instead of 655 amino acids) but also completely out of frame with no significant overlap with the wild type protein. The mutated product lacks the glycosidase hydrolase domain, the key domain of the protein^47,48^. In addition, expression levels were undetectable in the spleen consistent with nonsense-mediated decay^49^.

The second case of MPS I was in a dilute Boston Terrier with a similar clinical presentation and was also homozygous for the same *IDUA* mutation. This mutation was not found in 150 Boston Terrier samples in either the heterozygous or homozygous state indicating that its allele frequency is not very high in the general population of the breed. The two cases shared distinctive coat colors that are not due to their MPS disease. The AKC breed standard for the Boston Terrier disqualifies these coat colors. These two puppies both came from the southeastern United States and were purchased from breeders specializing in the disqualifying coat colors. The coat colors are recessive so in order to produce many puppies with these coat colors recent inbreeding likely occurred. While the allele frequency is low in the general population of Boston Terriers it is likely higher within the off color dogs.

IDUA enzyme deficiency is the known cause of MPS type I in humans^1^, mice^50^, cats^51^, and Plott Hound dogs^30^. Previous unpublished reports have added Boston Terriers to this growing list, however the causative variants were never identified in this breed^16^. Repeated reporting of MPS I in Boston Terriers suggests that this mutation could be in the breed, however the coat colors of the affected dogs were not reported. Thus, it is unknown whether this mutation exists in coat colors recognized by AKC. It is also possible that the breed has more than one mutation causing MPS. The insertion mutation is novel compared to the one established in Plott Hounds and explained by SNP mutation in the donor splice site^52,53^.

This study identified the causative mutation of a spontaneous canine model for human MPS I in the Boston Terriers. The identification of the causative variant will enable easier diagnosis of similar cases in the future and selective breeding to avoid the spread of this disease in the breed. Application of WGS to identify the causative mutation of MPS in Boston Terriers not only enriches our knowledge about the molecular mechanisms of this debilitating diseases but also augments recent studies to prove the efficiency of this technique in canine rare disease gene discovery^45,46^.

## Methods

### Ethics statement

The dogs are privately owned and informed consent was obtained from the dog owners for the participation of the dogs in the study. The study was approved by the UC Davis IACUC (Protocols # 18561 and 21084). Principal Investigator: Danika L. Bannasch. Institution: University of California, Davis. Active protocols are reviewed annually. This institution is accredited by the Association for Assessment and Accreditation of Laboratory Animal Care, International (AAALAC). This institution has an Animal Welfare Assurance on file with the Office of Laboratory Animal Welfare (OLAW). The Assurance Number is A3433-01. The IACUC is constituted in accordance with U.S. Public Health Service (PHS) Animal Welfare Policy and includes a member of the public and a non-scientist. The study was performed in accordance with relevant guidelines and regulations.

### Ophthalmic examination

An ophthalmic examination was performed using slit lamp biomicroscopy (SL-17 Slit-Lamp, Kowa Optimed, Inc., Torrance, CA, USA) and binocular indirect ophthalmoscopy (Vantage Plus Wireless, Keeler Instruments Inc., Broomall, PA, USA) using a 2.2 panretinal indirect lens (Volk Optical, Inc., Mentor, OH, USA). Tear production was evaluated with the Schirmer tear test (Merck Animal Health, Millsboro, DE) while intraocular pressure was assessed with rebound Tonometry (TonoVet; Icare Finland Oy, Espoo, Finland).

### Radiological assessment

X-ray studies were done using Summit Industries Model L177-01 (Summit Industries, 2901 W Lawrence Ave, Chicago, IL. 60625). Magnetic resonance imaging was done using 1.5-T scanner (General Electric Signa LX, Milwaukee, WI).

### Histopathological analysis

As described previously^54^, tissue sections were processed and embedded for routine paraffin sectioning. Tissue sections were cut at 4uM thickness on a rotary microtome (Leica), and stained for routine hematoxylin and eosin (H&E) using an autostainer (Dako). Serial, unstained sections were subsequently cut and stained with periodic acid-Schiff (PAS) or alcian blue methods. All slides were visualized with a clinical microscope (Olympus Bx-43) and representative images were obtained on an Olympus DP-72 camera using CellSens Entry acquisition software version 1.18, build16686 (Olympus)^55^.

### Urine GAGs analysis

For the first dog, urine spot test for MPS was performed by the University of Pennsylvania, School of Veterinary Medicine, PennGen testing laboratory and was reported as positive for MPS. For the second dog, urine GAGs were analyzed at the Stanford Clinical Biochemical Genetics Laboratory by quantitative assay (dimethylmethylene blue binding) and thin-layer chromatography.

### Whole genome sequencing

Frozen tissue was processed to extract 2 µg of high-quality input gDNA (Gentra Puregene DNA purification extraction kit, Qiagen, Valencia, CA). Qubit (Thermo Fisher Scientific Inc) was used for accurate DNA quantification using a fluorometric-based approach. DNA shearing was done using Covaris E220 sonicator followed by selection of 400 bp insert size fragments. PCR-free library prep kit was used to avoid PCR induced bias. Sequencing was performed in one lane on the Illumina HiSeq4000 platform in UC-Davis sequencing facility for 150 cycles in paired end mode.

### Bioinformatics analysis

A pipeline was implemented to identify the putatively deleterious protein coding variants. The process started by pre-processing of sequencing reads to remove sequencing adaptors and low quality sequences using Trimmomatic software package (V 0.36) “command line options: ILLUMINACLIP:$TRIM/adapters/TruSeq3-PE.fa:2:30:10:1 SLIDINGWINDOW:4:2 MINLEN:20”^56^. Orphan reads were excluded and only long enough paired-end reads (>20 bp) were kept. High quality reads were aligned to the UCSC dog reference genome using the BWA-MEM algorithm of BWA software package (v0.7.7) “command line options: -t 8 –M”^57^. BAM files were generated and sorted using SAMTools (v1.3.1)^58^ and indexed using Picard software (release 1.113)^59^. Following their best practice recommendations, GATK software package allowed additional step of base quality recalibration to adjust for possible systematic technical errors of sequencing quality scoring^60^. Base quality recalibration was done using a list of 11,034,217 known SNPs and 4,947,081 known INDELS generated by aggressive quality filtration of the 100 dog database of variants using the VariantFiltration tool of GATK to mark untrusted variants^60^ and the view tool of bcftools(v1.2) to exclude them out. Variant calling was done using GATK HaplotypeCaller (v3.5)^60^. Functional effects of variants were annotated using Variant Effect Predictor (VEP v85) made by Ensembl^61^. NCBI annotation was used for detection of genome errors. NCBI gene models are primarily based on RefSeq transcripts and thus overcome genome assembly errors. The NCBI sequences that do not match the reference assembly are annotated for possible assembly errors^62^. Phasing analysis of the deleterious heterozygous mutation was done using whatshap 0.18^63^.

### Sanger sequencing

PCR primers were designed to amplify the candidate variant in the *IDUA* gene (forward primer: GGAGCCCTCACCAGAAGC, and reverse primer AGACCACCCCTCCACAAAAG). The reaction included 14.24 μl water, 5 μl 5x KAPA LongRange Buffer, 1.5 μl MgCl_2_ (25 mM), 0.5 μl dNTP (10 mM), 0.63 μl of each forwa rd and reverse primers (20 μM), 0.25 μl of KAPA LongRange HotStart DNA  Polymerase (2.5U/µL) (Kapa Biosystems, Wilmington, MA, USA), 1.25 μl dimethyl sulfoxide **(**5%), and 1 μl of template genomic DNA. The reaction mixtures were subjected to a thermal cycling program of 95 °C for 5 min, followed by 35 cycles of 95 °C for 30 sec, 58 °C for 30 sec, and 72 °C for 1 min and final elongation stage of 72 °C for 1 min. The PCR products were purified using ExoSap (Thermo Fisher Scientific, Inc., Waltham, MA, USA) according to manufacturer's instructions and sequenced using the Sanger sequencing. Products were aligned to the UCSC genome browser (genome.ucsc.edu/).

### Semi-quantitative RT-PCR

RNA was extracted from the patient's spleen sample and a matching sample from another unaffected Boston Terrier’using RNeasy Plus Tissue Mini Kit (Qiagen, Valencia, CA, USA). cDNA was prepared using QuantiTect Reverse Transcription Kit (Qiagen, Valencia, CA, USA). The RT PCR reaction was performed using *IDUA* primers designed on Primer3 while *RPS5* primers were designed as recommended by Brinkhof *et al*. (Table 1)^64,65^. Each reaction included 14.3 μl water, 2 μl 5x Buffer with MgCl_2_, 1 μl dNTP (20 mM), 0.8 μl of each forward and reverse primers (20 μM), 0.1 μl of HotStarTaq. DNA Polymerase (Qiagen, Valenica, CA, USA), and 1 μl of cDNA made from1000 ng of RNA. Amplified products were visualized on a 2% agarose gel

**Table 1:** Primers used for semi-qPCR.

	Forward Primer (5’→3’)	Reverse Primer (5’→3’)
*IDUA*	AGCTCAACCTGGCCTATGTG	TCAGAGCAGGCGTCGTAGTA
*RPS5*	TCACTGGTGAGAACCCCCT	CCTGATTCACACGGCGTAG

## Supplementary information

Supplementary Information.

Supplementary Information 2.

Supplementary Information 3.

Supplementary Information 4.

Supplementary Information 5.

## Data Availability

Whole-genome sequencing files reported in this paper can be found in the NCBI Sequence Read Archive (SRA Bioproject no. PRJNA575092).
