# Supplementary material for: Whole genome sequencing for mutation discovery in a single case of lysosomal storage disease (MPS type 1) in the dog
Source: Sci Rep. 2020 Apr 16;10:6558. doi: 10.1038/s41598-020-63451-4 (PMC7162951; doi:10.1038/s41598-020-63451-4)
Supplement: Supplementary file 1 — Supplementary Information. [file 41598_2020_63451_MOESM1_ESM.docx]

**Whole genome sequencing for mutation discovery in a single case of lysosomal storage disease (MPS type 1) in the dog**

**Tamer A. Mansour, Kevin D. Woolard, Karen L. Vernau, Devin M. Ancona, Sara M. Thomasy, Lionel Sebbag, Bret A. Moore, Marguerite F. Knipe, Haitham A. Seada, Tina M. Cowan, Miriam Aguilar, C. Titus Brown, Danika L. Bannasch**

| **MPS subtype** | **Impaired**  **enzymes** | **Accumulated**  **GAGs** | **Dog breeds & Literature** |
| --- | --- | --- | --- |
| MPS I | alpha-L-iduronidase | dermatan sulfate  heparan sulfate | A litter of Plott hounds^63,64*^ |
| MPS II | iduronate sulfatase | dermatan sulfate  heparan sulfate | Labrador retriever^65^ |
| MPS IIIA | heparin N-sulfatase | heparan sulfate | Wirehaired Dachshunds^66^ and Huntaway dogs^67^ |
| MPS IIIB | alpha-N-acetyl-glucosaminidase | heparan sulfate | Schipperke breed^68^ |
| MPS VI | Arylsulfatase B | dermatan sulfate | Miniature Pinscher^69^, Miniature Schnauzer^70,71^, and Miniature Poodle breeds^69,70,72*^ |
| MPS VII | beta-glucuronidase | dermatan sulfate, keratan sulfate, chondroitin sulfate | Mixed breed dog^73^, German Shepherd^74^, and a litter of Brazilian Terriers ^75^ |

***Supplementary Table S1***: Primary literature for impaired enzymes and accumulated GAGs in different dog breeds. * Haskins et al ^76^ reported unpublished observations of MPS I in the Rottweiler and Boston Terrier breeds, and MPS VI in the Welsh Corgi and Chesapeake Bay retriever.References:

63 Shull, R. M. *et al.* Canine alpha-L-iduronidase deficiency. A model of mucopolysaccharidosis I. *Am J Pathol* **109**, 244-248 (1982).

64 Shull, R. M. *et al.* Morphologic and biochemical studies of canine mucopolysaccharidosis I. *Am J Pathol* **114**, 487-495 (1984).

65 Wilkerson, M. J., Lewis, D. C., Marks, S. L. & Prieur, D. J. Clinical and morphologic features of mucopolysaccharidosis type II in a dog: naturally occurring model of Hunter syndrome. *Vet Pathol* **35**, 230-233 (1998).

66 Fischer, A. *et al.* Sulfamidase deficiency in a family of Dachshunds: a canine model of mucopolysaccharidosis IIIA (Sanfilippo A). *Pediatr Res* **44**, 74-82, doi:10.1203/00006450-199807000-00012 (1998).

67 Jolly, R. D. *et al.* Mucopolysaccharidosis IIIA (Sanfilippo syndrome) in a New Zealand Huntaway dog with ataxia. *N Z Vet J* **48**, 144-148, doi:10.1080/00480169.2000.36181 (2000).

68 Ellinwood, N. M. *et al.* A model of mucopolysaccharidosis IIIB (Sanfilippo syndrome type IIIB): N-acetyl-alpha-D-glucosaminidase deficiency in Schipperke dogs. *J Inherit Metab Dis* **26**, 489-504 (2003).

69 Neer, T. M. *et al.* Clinical vignette. Mucopolysaccharidosis VI in a miniature pinscher. *J Vet Intern Med* **9**, 429-433 (1995).

70 Berman, L. F., P.; Stieger, K.; van Hoeven, M.; Ellinwood, N.M.; Henthorn, P.S.; et al. . in *Proceedings of the 2nd International Conference: Advances in Canine and Feline Genomics.*

71 Perez, M. L. *et al.* Mucopolysaccharidosis type VI in a juvenile miniature schnauzer dog with concurrent hypertriglyceridemia, necrotizing pancreatitis, and diabetic ketoacidosis. *Can Vet J* **56**, 272-277 (2015).

72 Jolly, R. D. *et al.* Mucopolysaccharidosis type VI in a Miniature Poodle-type dog caused by a deletion in the arylsulphatase B gene. *N Z Vet J* **60**, 183-188, doi:10.1080/00480169.2011.642791 (2012).

73 Haskins, M. E., Desnick, R. J., DiFerrante, N., Jezyk, P. F. & Patterson, D. F. Beta-glucuronidase deficiency in a dog: a model of human mucopolysaccharidosis VII. *Pediatr Res* **18**, 980-984 (1984).

74 Silverstein Dombrowski, D. C. *et al.* Mucopolysaccharidosis type VII in a German Shepherd Dog. *J Am Vet Med Assoc* **224**, 553-557, 532-553 (2004).

75 Hytonen, M. K. *et al.* A novel GUSB mutation in Brazilian terriers with severe skeletal abnormalities defines the disease as mucopolysaccharidosis VII. *PLoS One* **7**, e40281, doi:10.1371/journal.pone.0040281 (2012).

76 Haskins, M. E., Giger, U. & Patterson, D. F. in *Fabry Disease: Perspectives from 5 Years of FOS* (eds A. Mehta, M. Beck, & G. Sunder-Plassmann) (2006).
