# Supplementary material for: Whole genome sequencing for mutation discovery in a single case of lysosomal storage disease (MPS type 1) in the dog
Source: Sci Rep. 2020 Apr 16;10:6558. doi: 10.1038/s41598-020-63451-4 (PMC7162951; doi:10.1038/s41598-020-63451-4)

**Whole genome sequencing for mutation discovery in a single case of lysosomal storage disease (MPS type 1) in the dog**

**Tamer A. Mansour, Kevin D. Woolard, Karen L. Vernau, Devin M. Ancona, Sara M. Thomasy, Lionel Sebbag, Bret A. Moore, Marguerite F. Knipe, Haitham A. Seada, Tina M. Cowan, Miriam Aguilar, C. Titus Brown, Danika L. Bannasch**

**All possible alignments of the candidate insertion**

ATG--------CGGCCCCCCGGCCCCCGCGCCCCCGGGCTGGCGCTGCTGGCC (Reference sequence)

ATGCGGCCCCCCGGCCCCCCGGCCCCCGCGCCCCCGGGCTGGCGCTGCTGGCC (c.3_4insCGGCCCCC)

ATGC--------GGCCCCCCGGCCCCCGCGCCCCCGGGCTGGCGCTGCTGGCC (Reference sequence)

ATGCGGCCCCCCGGCCCCCCGGCCCCCGCGCCCCCGGGCTGGCGCTGCTGGCC (c.4_5insCGGCCCCC)

ATGCG--------GCCCCCCGGCCCCCGCGCCCCCGGGCTGGCGCTGCTGGCC (Reference sequence)

ATGCGGCCCCCCGGCCCCCCGGCCCCCGCGCCCCCGGGCTGGCGCTGCTGGCC (c.5_6insCGGCCCCC)

ATGCGG--------CCCCCCGGCCCCCGCGCCCCCGGGCTGGCGCTGCTGGCC (Reference sequence)

ATGCGGCCCCCCGGCCCCCCGGCCCCCGCGCCCCCGGGCTGGCGCTGCTGGCC (c.6_7insCGGCCCCC)

ATGCGGC--------CCCCCGGCCCCCGCGCCCCCGGGCTGGCGCTGCTGGCC (Reference sequence)

ATGCGGCCCCCCGGCCCCCCGGCCCCCGCGCCCCCGGGCTGGCGCTGCTGGCC (c.7_8insCGGCCCCC)

ATGCGGCC--------CCCCGGCCCCCGCGCCCCCGGGCTGGCGCTGCTGGCC (Reference sequence)

ATGCGGCCCCCCGGCCCCCCGGCCCCCGCGCCCCCGGGCTGGCGCTGCTGGCC (c.8_9insCGGCCCCC)

ATGCGGCCC--------CCCGGCCCCCGCGCCCCCGGGCTGGCGCTGCTGGCC (Reference sequence)

ATGCGGCCCCCCGGCCCCCCGGCCCCCGCGCCCCCGGGCTGGCGCTGCTGGCC (c.9_10insCGGCCCCC)

ATGCGGCCCC--------CCGGCCCCCGCGCCCCCGGGCTGGCGCTGCTGGCC (Reference sequence)

ATGCGGCCCCCCGGCCCCCCGGCCCCCGCGCCCCCGGGCTGGCGCTGCTGGCC (c.10_11insCGGCCCCC)

ATGCGGCCCCC--------CGGCCCCCGCGCCCCCGGGCTGGCGCTGCTGGCC (Reference sequence)

ATGCGGCCCCCCGGCCCCCCGGCCCCCGCGCCCCCGGGCTGGCGCTGCTGGCC (c.11_12insCGGCCCCC)

ATGCGGCCCCCC--------GGCCCCCGCGCCCCCGGGCTGGCGCTGCTGGCC (Reference sequence)

ATGCGGCCCCCCGGCCCCCCGGCCCCCGCGCCCCCGGGCTGGCGCTGCTGGCC (c.12_13insCGGCCCCC)

ATGCGGCCCCCCG--------GCCCCCGCGCCCCCGGGCTGGCGCTGCTGGCC (Reference sequence)

ATGCGGCCCCCCGGCCCCCCGGCCCCCGCGCCCCCGGGCTGGCGCTGCTGGCC (c.13_14insCGGCCCCC)

ATGCGGCCCCCCGG--------CCCCCGCGCCCCCGGGCTGGCGCTGCTGGCC (Reference sequence)

ATGCGGCCCCCCGGCCCCCCGGCCCCCGCGCCCCCGGGCTGGCGCTGCTGGCC (c.14_15insCGGCCCCC)

ATGCGGCCCCCCGGC--------CCCCGCGCCCCCGGGCTGGCGCTGCTGGCC (Reference sequence)

ATGCGGCCCCCCGGCCCCCCGGCCCCCGCGCCCCCGGGCTGGCGCTGCTGGCC (c.15_16insCGGCCCCC)

ATGCGGCCCCCCGGCC--------CCCGCGCCCCCGGGCTGGCGCTGCTGGCC (Reference sequence)

ATGCGGCCCCCCGGCCCCCCGGCCCCCGCGCCCCCGGGCTGGCGCTGCTGGCC (c.16_17insCGGCCCCC)

ATGCGGCCCCCCGGCCC--------CCGCGCCCCCGGGCTGGCGCTGCTGGCC (Reference sequence)

ATGCGGCCCCCCGGCCCCCCGGCCCCCGCGCCCCCGGGCTGGCGCTGCTGGCC (c.17_18insCGGCCCCC)

ATGCGGCCCCCCGGCCCC--------CGCGCCCCCGGGCTGGCGCTGCTGGCC (Reference sequence)

ATGCGGCCCCCCGGCCCCCCGGCCCCCGCGCCCCCGGGCTGGCGCTGCTGGCC (c.18_19insCGGCCCCC)

ATGCGGCCCCCCGGCCCCC--------GCGCCCCCGGGCTGGCGCTGCTGGCC (Reference sequence)

ATGCGGCCCCCCGGCCCCCCGGCCCCCGCGCCCCCGGGCTGGCGCTGCTGGCC (c.19_20insCGGCCCCC)

**Reference mRNA sequence**

https://www.ncbi.nlm.nih.gov/nuccore/926234517?report=fasta

>NM_001313883.1 Canis lupus familiaris iduronidase, alpha-L- (IDUA), mRNA (the coding sequence is highlighted in yellow)

CGCGACCCCCGCCCGGCCATGCGGCCCCCCGGCCCCCGCGCCCCCGGGCTGGCGCTGCTGGCCGCGCTGC TGGCGGCGCCCCGGGCCCTCGCAGAGGCCCCGCACCTGGTGCTCGTGGACGCGGCCCGCGCGCTGCGGCC CCTGCGGCCCTTCTGGAGGAGCACCGGCTTCTGCCCCCCCCTGCCGCACAGCCAGGCTGACCGCTATGAC CTCAGCTGGGACCAGCAGCTCAACCTGGCCTATGTGGGTGCTGTCCCTCACGGGGGCATCGAGCAGGTCC GGACCCACTGGCTGCTGGAGCTCATCACGGCCAGGGAGTCAGCTGGGCAAGGCCTGAGCTACAACTTCAC CCACCTGGATGGCTACCTGGATCTCCTCAGGGAGAACCAGCTCCTCCCAGGTTTTGAGCTGATGGGCAGC CCCTCCCAGCGCTTCACCGACTTCGAGGACAAGCGGCAGGTGTTGGCGTGGAAGGAGCTGGTGTCCCTCC TGGCCAGGAGATACATCGGGAGGTATGGACTCTCATACGTTTCCAAGTGGAACTTCGAGACATGGAATGA GCCAGACCACCACGACTTCGACAACGTGACCATGACCCTGCAAGGCTTCCTGAACTACTACGACGCCTGC TCTGAGGGTCTGCGTGCTGCCAGCCCGGCCCTGCGCCTTGGCGGCCCCGGGGACTCTTTCCACCCCTGGC CGCGCTCCCCCCTGTGCTGGGGCCTCCTGGAGCATTGTCACAACGGCACCAACTTCTTCACCGGGGAGCT GGGGGTGCGCCTGGACTACATCTCCCTCCACAAGAAGGGCGCGGGGAGCTCCATCTACATCCTGGAACAG GAGCAGGCCACCGTGCAGCAGATCCGACGGCTCTTCCCCAAGTTCGCCGACACCCCCGTTTACAACGACG AGGCGGACCCGCTGGTGGGCTGGGCCCTGCCGCAGCCCTGGAGAGCCGACGTGACGTACGCGGCCATGGT GGTGAAGGTCGTGGCGCAGCACCAGAACCCGCCCCGGGCCAACGGCAGCGCGGCCCTGCGCCCCGCGCTC CTGAGCAACGACAACGCCTTCCTGAGCTTCCACCCGCACCCGTTCACGCAGCGCACGCTCACCGCGCGCT TCCAGGTCAACGACACGGAGCCGCCGCACGTGCAGCTGCTGCGCAAGCCGGTGCTCACGGCCATGGCGCT GCTGGCCCTGCTGGACGGCCGGCAGCTGTGGGCCGAGGTGTCGCGGGGCGGGACGGTGCTGGACAGCAAC CACACGGTGGGCGTCCTGGCCAGCGCGCACCTGCCGGCCGGGCCCCGGGACGCCTGGCGCGCCACCGTGC TGCTCTACGCGAGCGACGACACGCGCGCCCACGCCGCCCGCGCCGTGCCCGTGACGCTGCGCCTGCTCGG GGTGCCGCGGGGCCCAGGGCTCGTCTACGTCACCCTGGCCCTGGACAACCCGCGCTGCAGCCCCCACCGC GAGTGGCAGCGCCTGAGCCGGCCCGTCTTCCCCACGGCGGAGGAGTTCCGGCGCATGCGCGCAGCCGAGG ACCCGGTGGCCGAGGCGCCGCGCCCCTTCCCCGCCAGCGGCCGCCTGACGCTCAGCGTGGAGCTGCGGCT GCCCTCGCTGCTGCTGCTGCACGTGTGCGCGCGCCCGGAGAAGCCGCCGGGACCGGTGACCCGGCTCCGT GCCCTGCCCTTGACCCGTGGGCAGGTGCTTTTGGTGTGGTCGGATGAGCGCGTGGGCTCCAAGTGCCTGT GGACCTATGAGATCCAGTTCTCCGCGGATGGAGAAGTGTACACGCCCATCAGCAGGAAGCCATCCACCTT TAACCTGTTTGTGTTCAGCCCAGAGTCAGCCGTCACCTCTGGCTCCTACCGGGTTCGAGCGGTGGACTAC TGGGCCCGACCGGGCCCCTTCTCGACCCGCGTGCACTACGTGGAGGTCCCTGCACCGTCAGGGCCGCCGC GGCCCAGTGACTGTGAGCGGTGCTGA

**MPS dog mRNA sequence**

>IDUA_in_MPS_dog, mRNA (coding sequence of the 1st open reading frame is highlighted in yellow and the insertion sequence is red) CGCGACCCCCGCCCGGCCATGCGGCCCCCCGGCCCCC**CGGCCCCC**GCGCCCCCGGGCTGGCGCTGCTGGCCGCGCTGC TGGCGGCGCCCCGGGCCCTCGCAGAGGCCCCGCACCTGGTGCTCGTGGACGCGGCCCGCGCGCTGCGGCC CCTGCGGCCCTTCTGGAGGAGCACCGGCTTCTGCCCCCCCCTGCCGCACAGCCAGGCTGACCGCTATGAC CTCAGCTGGGACCAGCAGCTCAACCTGGCCTATGTGGGTGCTGTCCCTCACGGGGGCATCGAGCAGGTCC GGACCCACTGGCTGCTGGAGCTCATCACGGCCAGGGAGTCAGCTGGGCAAGGCCTGAGCTACAACTTCAC CCACCTGGATGGCTACCTGGATCTCCTCAGGGAGAACCAGCTCCTCCCAGGTTTTGAGCTGATGGGCAGC CCCTCCCAGCGCTTCACCGACTTCGAGGACAAGCGGCAGGTGTTGGCGTGGAAGGAGCTGGTGTCCCTCC TGGCCAGGAGATACATCGGGAGGTATGGACTCTCATACGTTTCCAAGTGGAACTTCGAGACATGGAATGA GCCAGACCACCACGACTTCGACAACGTGACCATGACCCTGCAAGGCTTCCTGAACTACTACGACGCCTGC TCTGAGGGTCTGCGTGCTGCCAGCCCGGCCCTGCGCCTTGGCGGCCCCGGGGACTCTTTCCACCCCTGGC CGCGCTCCCCCCTGTGCTGGGGCCTCCTGGAGCATTGTCACAACGGCACCAACTTCTTCACCGGGGAGCT GGGGGTGCGCCTGGACTACATCTCCCTCCACAAGAAGGGCGCGGGGAGCTCCATCTACATCCTGGAACAG GAGCAGGCCACCGTGCAGCAGATCCGACGGCTCTTCCCCAAGTTCGCCGACACCCCCGTTTACAACGACG AGGCGGACCCGCTGGTGGGCTGGGCCCTGCCGCAGCCCTGGAGAGCCGACGTGACGTACGCGGCCATGGT GGTGAAGGTCGTGGCGCAGCACCAGAACCCGCCCCGGGCCAACGGCAGCGCGGCCCTGCGCCCCGCGCTC CTGAGCAACGACAACGCCTTCCTGAGCTTCCACCCGCACCCGTTCACGCAGCGCACGCTCACCGCGCGCT TCCAGGTCAACGACACGGAGCCGCCGCACGTGCAGCTGCTGCGCAAGCCGGTGCTCACGGCCATGGCGCT GCTGGCCCTGCTGGACGGCCGGCAGCTGTGGGCCGAGGTGTCGCGGGGCGGGACGGTGCTGGACAGCAAC CACACGGTGGGCGTCCTGGCCAGCGCGCACCTGCCGGCCGGGCCCCGGGACGCCTGGCGCGCCACCGTGC TGCTCTACGCGAGCGACGACACGCGCGCCCACGCCGCCCGCGCCGTGCCCGTGACGCTGCGCCTGCTCGG GGTGCCGCGGGGCCCAGGGCTCGTCTACGTCACCCTGGCCCTGGACAACCCGCGCTGCAGCCCCCACCGC GAGTGGCAGCGCCTGAGCCGGCCCGTCTTCCCCACGGCGGAGGAGTTCCGGCGCATGCGCGCAGCCGAGG ACCCGGTGGCCGAGGCGCCGCGCCCCTTCCCCGCCAGCGGCCGCCTGACGCTCAGCGTGGAGCTGCGGCT GCCCTCGCTGCTGCTGCTGCACGTGTGCGCGCGCCCGGAGAAGCCGCCGGGACCGGTGACCCGGCTCCGT GCCCTGCCCTTGACCCGTGGGCAGGTGCTTTTGGTGTGGTCGGATGAGCGCGTGGGCTCCAAGTGCCTGT GGACCTATGAGATCCAGTTCTCCGCGGATGGAGAAGTGTACACGCCCATCAGCAGGAAGCCATCCACCTT TAACCTGTTTGTGTTCAGCCCAGAGTCAGCCGTCACCTCTGGCTCCTACCGGGTTCGAGCGGTGGACTAC TGGGCCCGACCGGGCCCCTTCTCGACCCGCGTGCACTACGTGGAGGTCCCTGCACCGTCAGGGCCGCCGC GGCCCAGTGACTGTGAGCGGTGCTGA

**Translated proteins from wild type IDAU gene**

http://www.uniprot.org/uniprot/F1P608.fasta

>tr|F1P608|F1P608_CANLF Alpha-L-iduronidase OS=Canis lupus familiaris GN=IDUA PE=4 SV=2 MRPPGPRAPGLALLAALLAAPRALAEAPHLVLVDAARALRPLRPFWRSTGFCPPLPHSQA DRYDLSWDQQLNLAYVGAVPHGGIEQVRTHWLLELITARESAGQGLSYNFTHLDGYLDLL RENQLLPGFELMGSPSQRFTDFEDKRQVLAWKELVSLLARRYIGRYGLSYVSKWNFETWN EPDHHDFDNVTMTLQGFLNYYDACSEGLRAASPALRLGGPGDSFHPWPRSPLCWGLLEHC HNGTNFFTGELGVRLDYISLHKKGAGSSIYILEQEQATVQQIRRLFPKFADTPVYNDEAD PLVGWALPQPWRADVTYAAMVVKVVAQHQNPPRANGSAALRPALLSNDNAFLSFHPHPFT QRTLTARFQVNDTEPPHVQLLRKPVLTAMALLALLDGRQLWAEVSRGGTVLDSNHTVGVL ASAHLPAGPRDAWRATVLLYASDDTRAHAARAVPVTLRLLGVPRGPGLVYVTLALDNPRC SPHREWQRLSRPVFPTAEEFRRMRAAEDPVAEAPRPFPASGRLTLSVELRLPSLLLLHVC ARPEKPPGPVTRLRALPLTRGQVLLVWSDERVGSKCLWTYEIQFSADGEVYTPISRKPST FNLFVFSPESAVTSGSYRVRAVDYWARPGPFSTRVHYVEVPAPSGPPRPSDCERC*

**Translated proteins from mutated IDAU gene**

http://web.expasy.org/cgi-bin/translate/dna_aa

>IDUA_in_MPS_dog, ptn (5’3’ Frame 1) (1^st^ ORF is highlighted in yellow)

MRPPGPPAPAPPGWRCWPRCWRRPGPSQRPRTWCSWTRPARCGPCGPSGGAPASAPPCRT

ARLTAMTSAGTSSSTWPMWVLSLTGASSRSGPTGCWSSSRPGSQLGKA*ATTSPTWMATW

ISSGRTSSSQVLS*WAAPPSASPTSRTSGRCWRGRSWCPSWPGDTSGGMDSHTFPSGTSR

HGMSQTTTTSTT*P*PCKAS*TTTTPALRVCVLPARPCALAAPGTLSTPGRAPPCAGASW

SIVTTAPTSSPGSWGCAWTTSPSTRRARGAPSTSWNRSRPPCSRSDGSSPSSPTPPFTTT

RRTRWWAGPCRSPGEPT*RTRPWW*RSWRSTRTRPGPTAARPCAPRS*ATTTPS*ASTRT

RSRSARSPRASRSTTRSRRTCSCCASRCSRPWRCWPCWTAGSCGPRCRGAGRCWTATTRW

ASWPARTCRPGPGTPGAPPCCSTRATTRAPTPPAPCP*RCACSGCRGAQGSSTSPWPWTT

RAAAPTASGSA*AGPSSPRRRSSGACAQPRTRWPRRRAPSPPAAA*RSAWSCGCPRCCCC

TCARARRSRRDR*PGSVPCP*PVGRCFWCGRMSAWAPSACGPMRSSSPRMEKCTRPSAGS

HPPLTCLCSAQSQPSPLAPTGFERWTTGPDRAPSRPACTTWRSLHRQGRRGPVTVSGA

**BlastP alignment**

Wild type IDUA protein is the subject and the truncated 108 amino acid product in the MPS dog in the query


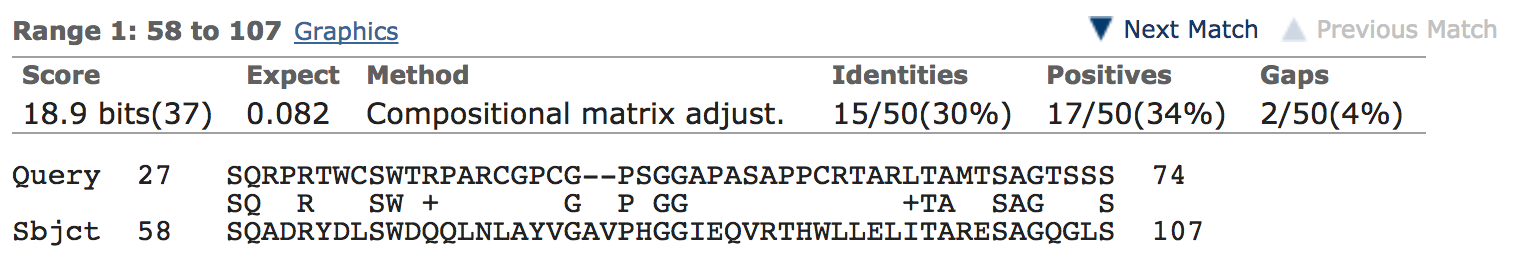


**Protein domain summary of Ensembl**

The graph shows the full length of IDUA gene (ENSCAFP00000024558.3) along its 655 as observed on the scale bar. The tracks recognize the know protein domains of IDUA in several databases: hmmpanther, Superfamily domains, Prints domain, Pfam domain, PROSITE patterns, and Gene 3D


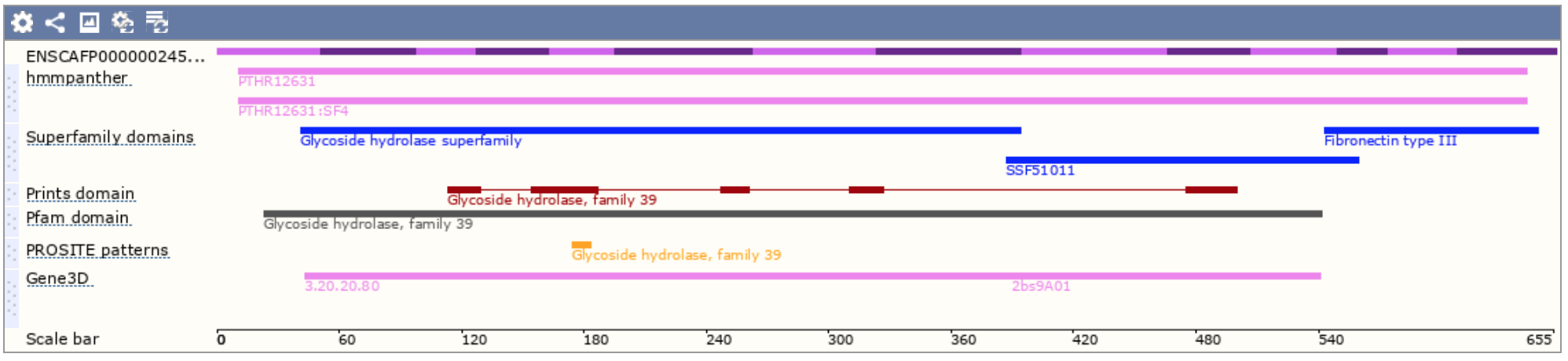


**Full-length gel for the cropped images in Figure 3B:** Semi-qRT-PCR of *IDUA* and *RPS5* expression in spleen samples from affected and unaffected Boston Terriers


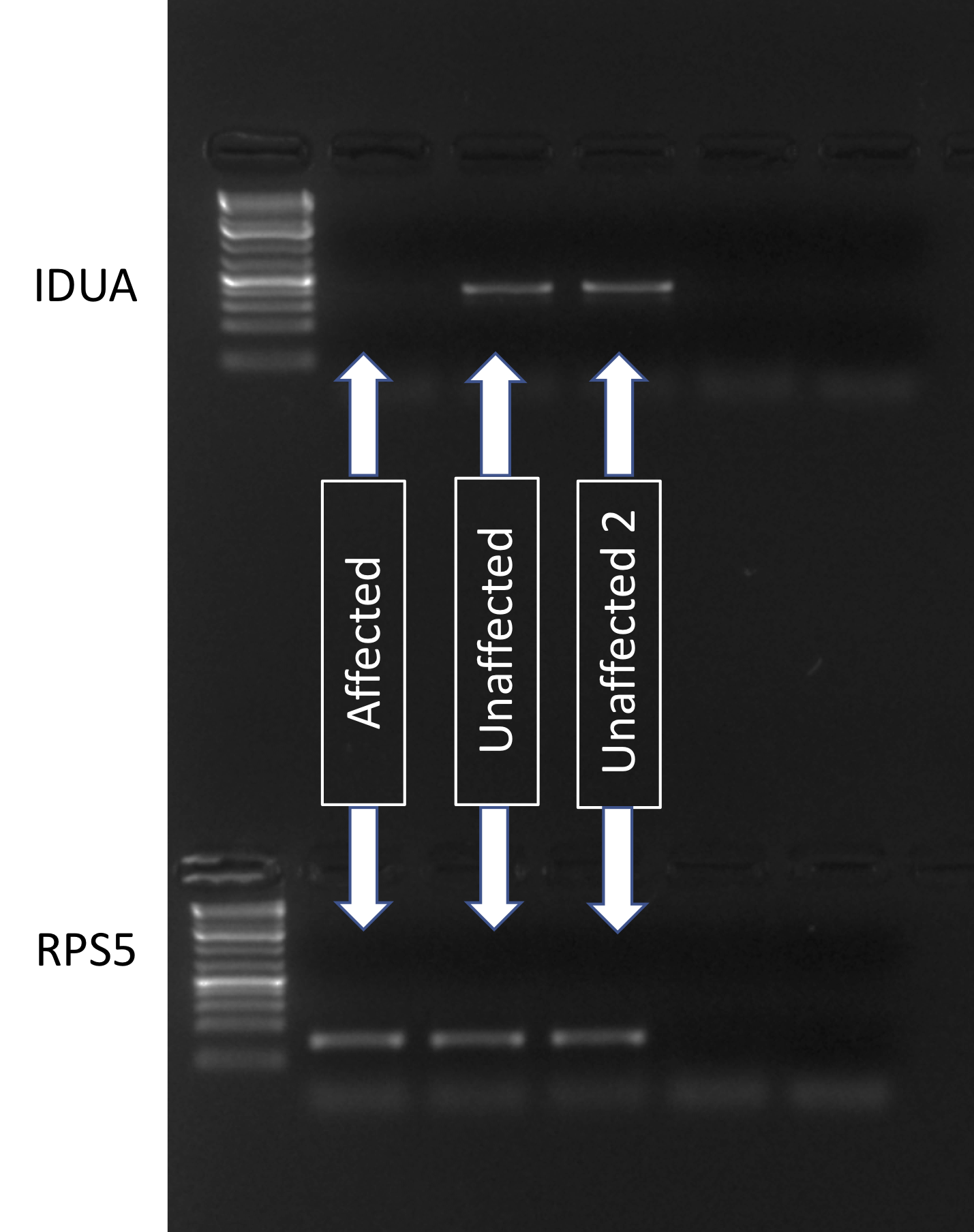

Supplement: Supplementary file 5 — Supplementary Information 5. [file 41598_2020_63451_MOESM5_ESM.docx]
